# Whole body irradiation and agonist anti-CD40 synergize to promote adoptive T cell therapy of resistant murine pancreatic neuroendocrine tumors

**DOI:** 10.1186/2051-1426-3-S2-P50

**Published:** 2015-11-04

**Authors:** Lindsay Ward-Kavanagh, Timothy K Cooper, Aron Lukacher, Todd D Schell

**Affiliations:** 1 Penn State Hershey College of Medicine, Hershey, PA, USA

## Background

Adoptive T cell therapy (ACT) can promote dramatic regressions of established cancer, yet durable clinical responses are observed in only a subset of patients. ACT provides the opportunity to alter the immune suppressive host environment associated with cancer prior to introduction of tumor-specific T cells.

## Methods

Here, we evaluated the utility of combined whole body irradiation and agonist anti-CD40 antibody to enhance ACT-mediated control of established autochthonous pancreatic tumors.

## Results

Sublethal whole body irradiation, a conditioning regimen associated with donor T cell persistence, had little impact on donor T cell magnitude or disease outcome, but did increase T cell persistence. Anti-CD40 conditioning, an approach known to enhance antigen presenting cell function and T cell expansion, transiently increased T cell accumulation in the lymphoid organs and the pancreas, but failed to eliminate established disease. In contrast, combined whole body irradiation and anti-CD40 prolonged T cell proliferation in the tumor draining lymph node and dramatically increased accumulation of interferon gamma-producing, PD-1^lo^ donor T cells in the pancreas. Dual-conditioning with ACT also induced histologic regression of established tumors and significantly increased overall survival. Increased lifespan was entirely dependent upon T cell transfer, and partially dependent upon interferon gamma production by donor T cells.

## Conclusions

Our results identify the novel combination of two clinically relevant host conditioning approaches that together with ACT overcome tumor-induced immune suppression to produce dramatic therapeutic benefits against established tumors.

